# Mapping Drug Physico-Chemical Features to Pathway Activity Reveals Molecular Networks Linked to Toxicity Outcome

**DOI:** 10.1371/journal.pone.0012385

**Published:** 2010-08-27

**Authors:** Philipp Antczak, Fernando Ortega, J. Kevin Chipman, Francesco Falciani

**Affiliations:** School of Biosciences, University of Birmingham, Birmingham, United Kingdom; Memorial Sloan-Kettering Cancer Center, United States of America

## Abstract

The identification of predictive biomarkers is at the core of modern toxicology. So far, a number of approaches have been proposed. These rely on statistical inference of toxicity response from either compound features (i.e., QSAR), in vitro cell based assays or molecular profiling of target tissues (i.e., expression profiling). Although these approaches have already shown the potential of predictive toxicology, we still do not have a systematic approach to model the interaction between chemical features, molecular networks and toxicity outcome. Here, we describe a computational strategy designed to address this important need. Its application to a model of renal tubular degeneration has revealed a link between physico-chemical features and signalling components controlling cell communication pathways, which in turn are differentially modulated in response to toxic chemicals. Overall, our findings are consistent with the existence of a general toxicity mechanism operating in synergy with more specific single-target based mode of actions (MOAs) and provide a general framework for the development of an integrative approach to predictive toxicology.

## Introduction

One of the most challenging tasks in toxicology is the identification of a potential toxicity via high-throughput screening, avoiding the use of animals, at an early stage in the development programme of a product such as a pharmaceutical or in the context of REACH [1]. Such screens can help to reduce attrition of products late in development and can help to prioritise existing chemicals for more complete safety assessment. In this context, the concept of quantitative structure activity relationship (QSAR) analysis was originally developed with the purpose of predicting a toxicity or pharmacological response utilizing information on the physico-chemical features (PCFs) of a chemical and the relationship to biological effects. In the last 20 years QSAR analysis has been characterized by an increasing level of sophistication as technological and computational developments have made it possible to measure or compute a higher number of chemical and physical parameters [2]. In addition, recent reports have shown that the prediction accuracy of QSAR models can be increased when additional information from cell based assays is utilized [3], [4] Independently to these developments, the availability of functional genomics technologies facilitated the measurement of mRNA concentrations, proteins and metabolites in single experiments. This, together with the development of novel computational methods suitable for the analysis and integration of very large multilevel datasets [5], have contributed to demonstrate the usefulness of molecular fingerprinting in predicting toxicity from an early readout of the response to chemical exposure [5]–[9]. Toxicants can in some cases be discriminated according to their mechanism of action and their target organs [10], [11]. However, there have been no successful attempts to model the interaction between a drug PCFs with genome wide molecular response to exposure and put this in context with toxicity response. It is therefore still unclear whether a true integration between traditional QSAR and functional genomics data may be possible. In this paper we describe an analysis strategy which addresses this issue by integrating gene expression profiling measurements in the logical framework of QSAR analysis. We have applied this approach to a publicly available expression profiling dataset, representing the pre-phenotypic transcriptional response to chemical exposure in a rat model of renal tubular degeneration which is a major toxicological response contributing to attrition during drug development [12]. Our approach has successfully linked a sub-set of PCFs to the activity of signalling pathways known from the literature to drive effector pathways differentially modulated between toxic and non-toxic chemicals. This finding suggests the existence of general toxicity mechanisms which operate in synergy with specific single-target based MOAs. The approach we have used has general validity since it can be applied to integrate different types of PCFs, molecular and phenotypic measurements to identify predictors of toxicity within a mechanistic framework for biological interpretation.

## Results

### Rational of the approach and data analysis overview

**Figure 1:**
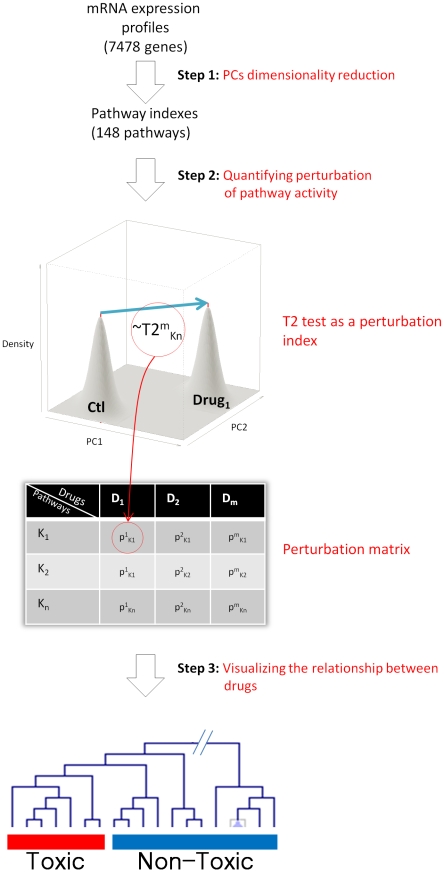
Analysis strategy to compute indices of pathway activity. To compute the indices of pathway activity the first step is to summarize the gene expression profiles using PCA according to KEGG pathways. This results in 148 pathway indices summarized using two PCs. These PC can then be used as an input to a 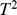 Hotelling's statistics to compute the perturbation index for a specific drug as compared to a matched control group. The third step is to visualize the relationship between the drugs with the use of a hierarchical clustering. We can then show that the dimensionality reduction in step 1 is biologically relevant to use in the subsequent analysis.

**Figure 2:**
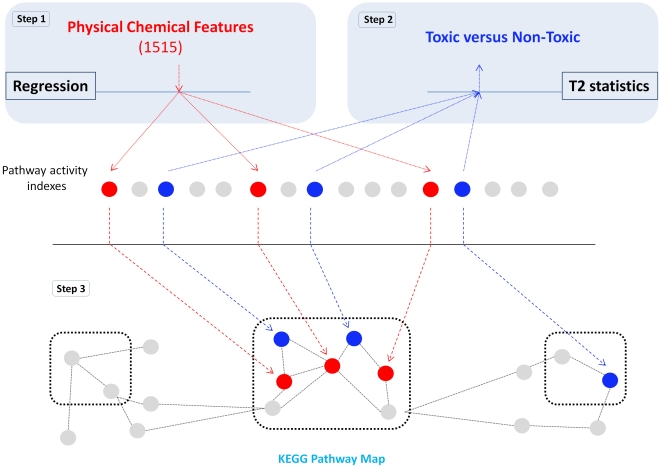
Integrating pathways associated to PCFs and toxicity. Alongside using a regression based model to identify pathways associated to PCFs (Step 1) we also identified pathways associated to toxicity by the use of the 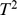 statistics (Step 2). The resulting pathways were then mapped onto a KEGG pathway map to identify clusters of pathways associated to both PCFs and toxicity (Step 3). Finally we asked the question if the PCFs we have found to be associated to pathways are a better predictor of toxicity.

The dataset we have used in this analysis is based on a wide range of chemicals. Some of them are known to work by different mechanisms of action and have diverse chemical structures. Despite this heterogeneity it has been shown that it is possible to identify early molecular response signatures predictive of in vivo toxicity outcome [13]. So far, it is unclear whether these signatures represent an early convergence of the different drugs MOAs towards common toxicity pathways or whether a component of them may represent a direct interaction between the chemicals and cellular components. Here we address this question by using a multi-step computational approach. Firstly, we simplify the complexity of the transcriptional response by computing indices of overall pathway transcriptional activity (Figure 1, Step 1). This effectively reduces the dataset from thousands of individual gene expression profiles to 148 pathway indices. We demonstrate that toxic and non-toxic chemicals can be separated on the basis of their ability to modify pathway activity (Figure 1, Step 2 and 3). This proves the biological relevance of the pathway indices. We then hypothesize that the defined set of a drug PCFs may be representative of the ability of a chemical to induce changes in the homeostatic state of the target organ. In line with this hypothesis we search for statistical models based on combinations of PCFs and predictive of the transcriptional response to drug exposure (Figure 2, Step 1). In parallel we identified which pathways are differentially modulated between samples treated with toxic and non-toxic drugs (Figure 2, Step 2). If these two pathway subsets truly represent the interaction between chemicals and underlying molecular networks we may expect that they would be part of a super-pathway. This hypothesis was addressed by mapping pathways in the two on the KEGG pathway map and testing for statistical association (Figure 2, Step 3).

### Computing indices of molecular pathway activity

The overall aim of this study was to link PCFs to drug-induced molecular responses and phenotypic outcome. A key challenge in identifying subsets of PCFs predictive of transcriptional response is the astronomical number of possible combinations of PCFs and gene subsets that need to be tested within a statistical modelling framework. In order to address this challenge we first simplified the complexity of the dataset by reducing thousands of individual gene expression profiles to a relatively small number of overall pathway activity indices. This was achieved by summarizing gene expression profiles representative of a given KEGG pathway with the first two principal components (PCs) of the gene expression matrix [14]. The choice of the number of PCs to construct the pathway activity indices was driven by the simple criteria to represent at least 80% of the variance present in the original dataset. By using this strategy we built a new dataset representing 148 KEGG Pathways (44% of the KEGG pathway database). This dataset represents 1676 out of the 7478 genes which were originally present in the processed Iconix dataset. We found that the apparent loss of gene representation was largely (77%) associated to the high frequency of non-annotated genes (i.e. function unknown or estimated by sequence homology). KEGG Pathways represented in the derived dataset are a good representation of the spectrum of functions covered by the KEGG database (Table S1 and S2 for a detailed breakdown in the functional representation of the KEGG pathways represented in the dataset).

### Molecular pathway activity in response to chemical exposure is correlated to toxicity

**Figure 3:**
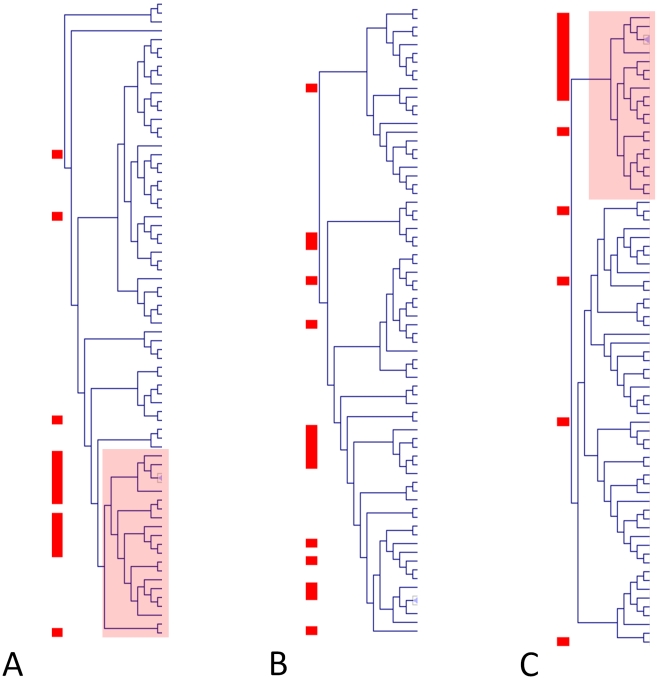
Hierarchical clustering of chemicals based on pathway modulation profiles. The figure shows the clustering of chemicals on the basis of the extent of change of transcriptional activity in molecular pathways after exposure. Panel A represent the relationship between the samples when the change in pathway activity is represented simultaneously by the first and second PCs (multi-variate 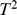 Hotelling test). Panels B and C represent respectively the results of cluster analysis when the change in pathway activity is estimated by the PC1 or the PC2 (univariate t-test). Notice that toxic chemicals cluster (highlighted areas on Panels A and C) on the basis of the multivariate test and that the information associated to toxicity is primarily represented by the PC2. Toxic chemicals have been highlighted using a red square on the left of each clustering.

In their original paper, Fielden et al [13] demonstrated that using statistical modelling techniques it is possible to identify subsets of genes predictive of late toxicity outcome. Since our strategy is based on simplifying the complexity of the data using indices of pathway activity we first asked whether these were also effective indicators of toxicity response. We first approached this question by clustering the chemicals on the basis of their ability to modify the transcriptional activity of a given pathway. Figure 3A shows that the profile of pathway perturbation is indeed informative of chemical toxicity. In particular, cluster analysis succeeded in grouping 12 out of 15 nephrotoxic chemicals within a well-defined cluster (Figure 3A). Analysis of the individual PCs revealed that the second PC on its own was sufficient to reproduce clustering of toxic chemicals without significant loss of information (Figure 3B and 3C). In order to identify the molecular pathways differentially modulated in response to toxic chemicals we directly compared the index of pathway activity between samples treated either with nephrotoxic or non-nephrotoxic chemicals. This analysis identified 21 pathways which were differentially modulated (FDR
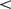
1%, Table 1). These can be grouped into three main functional categories: 1) metabolic pathways such as *Glycerophospholipid metabolism* or *Aminosugar metabolism*, 2) pathways with a strong signalling component such as *Parkinson's disease*, *Phosphatidylinositol signalling* and *Prostate cancer* and 3) cell communication pathways such as *Cell communication* and *Focal adhesion*. The KEGG pathway terms *Parkinson's disease*, *Prostate cancer*, *Pancreatic cancer* and *Renal cell carcinoma* do not specifically include the term ‘signalling’ in their definition but are indeed representing primarily signalling pathways. More specifically, the pathway *Parkinson's disease* represents the molecular events downstream dopamine stimulation, which is a major player in synaptic transmission and it is effectively linked to signalling pathways controlling vasoconstriction. This pathway is important for kidney physiology where dopamine release induce an increase in renal blood flow, urinary volume and excretion of sodium and potassium. This then leads to an increase in glomerular filtration rate as well as a depletion of plasma cyclic AMP [15]. The pathway *Prostate cancer* represents components of the *MAPK signalling* and *p53 signalling pathways* which are included in the response downstream of cytokine stimulation. Specific signalling pathways associated to the *Pancreatic cancer* pathway are *ErbB*, *Jak-STAT*, *VEGF*, *TGF-* 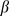, *MAPK* and the *p53 signalling pathway*. These are not only relevant to the biology of cancer (alteration in these signalling pathways destabilize growth inhibition and promote tumour growth activity [16]) but also to kidney response to stress and regeneration [17].

**Table 1:** This table shows 21 KEGG pathways that were found to be significantly perturbed by nephrotoxic chemicals (FDR
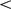
1%).

KEGG ID	Pathway Name	Size	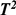 Score
rno05020	Parkinson's disease	10	31.71
rno00564	Glycerophospholipid metabolism	25	31.29
rno05215	Prostate cancer	52	26.66
rno02010	ABC transporters - General	13	26.35
rno04070	Phosphatidylinositol signaling system	32	22.56
rno04130	SNARE interactions in vesicular transport	24	21.54
rno00760	Nicotinate and nicotinamide metabolism	10	21.17
rno01430	Cell Communication	45	21.07
rno00530	Aminosugars metabolism	9	20.77
rno04120	Ubiquitin mediated proteolysis	65	20.40
rno05030	Amyotrophic lateral sclerosis (ALS)	16	19.64
rno03010	Ribosome	26	19.54
rno03050	Proteasome	20	19.35
rno05212	Pancreatic cancer	50	19.21
rno00230	Purine metabolism	64	16.14
rno04330	Notch signaling pathway	20	15.31
rno01031	Glycan structures - biosynthesis 2	17	14.96
rno04612	Antigen processing and presentation	23	14.96
rno04510	Focal adhesion	102	14.49
rno04320	Dorso-ventral axis formation	17	14.32
rno05211	Renal cell carcinoma	52	13.71

The number of genes in each pathway and the value of the 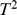 hotelling statistics are shown respectively in the third and fourth columns size column.

### Chemical features are predictive of molecular pathway activity

**Figure 4:**
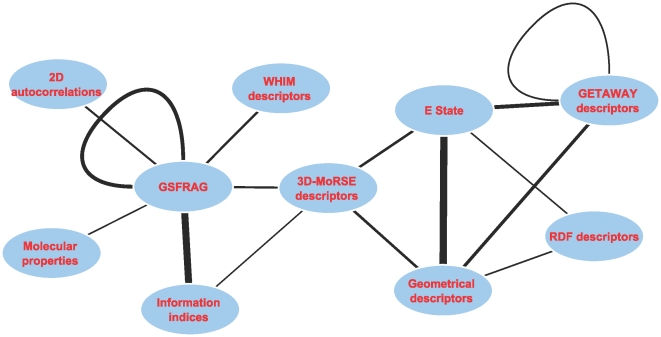
Visual summary of descriptor connections. The network represents the number of interactions between PCFs descriptor groups computed from pooled models. The thickness of the line is proportional to the number of pathways in which PCFs of a given descriptor group are selected in an interaction component of a predictive models. The highest value edge is found between ET-State and Geometrical descriptors in which 11 out of 19 pathways were found to contain models based on features from these 2 descriptor groups.

Having demonstrated that indices of pathway activity are representative of the biological effect of chemicals we addressed the hypothesis that a subset of PCFs may be correlated to the kidney transcriptional response to drug exposure. The statistical framework we have used to address this hypothesis (described in detail in the methods section) relies on a regression model explaining the activity of a given pathway (which we remind is the first or second principal component computed from the gene expression matrix associated to a given pathway) as a linear combination of three chemical features. The model also includes interaction components to take into account potential synergistic effects between chemical descriptors. We successfully identified predictive models (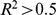) for 19 of the 148 pathways represented in the Iconix dataset. It is worth noticing that, pathway activity indices (Figure 3) as well as the original gene expression data (Figure S1), separates toxic from non-toxic chemicals across the second PC whereas the first component is likely to represent non-specific effects (Figure S1). Therefore the association between PCFs and the pathway activity indices build using the second component is biologically reasonable. Among pathways associated to chemical features we observed a large number of signalling pathways as well as some metabolic pathways (i.e. *Glycolysis*, *Porphyrin metabolism*, *Chlorophyll metabolism* and *Glutathione metabolism*). Two of these pathways were also found to be associated to toxicity in the analysis described in the previous paragraph (*Prostate cancer* and *Cell communication*). PCFs selected in the models could be assigned to several descriptor groups. Figure 4 summarizes in a graph format the most frequent combination of features' descriptors groups selected in the chemical feature models. A key feature of the selected models is the importance of interaction components which in most cases explain an average of 50% of the model variance (Figure S2). Descriptor groups pairs such as descriptors that describe patterns in the connection of specific atoms with each other (ET-State) and Geometrical descriptors or descriptors of special fragments that describe a path or cycle (GSFRAG) with itself are predominantly chosen by our method. All these descriptors classes capture different types of structural information. For example, GSFRAG descriptors identify specific chemical motives such as the size of a ring, or the length of linear connections; ET-States descriptors describe patterns in the connection of specific atoms with each other and geometrical descriptors are designed to capture patterns in the overall topology of the molecule.

### Pathways whose activity is correlated to chemical features are part of a signalling system closely connected with cellular communication and related functions

**Figure 5:**
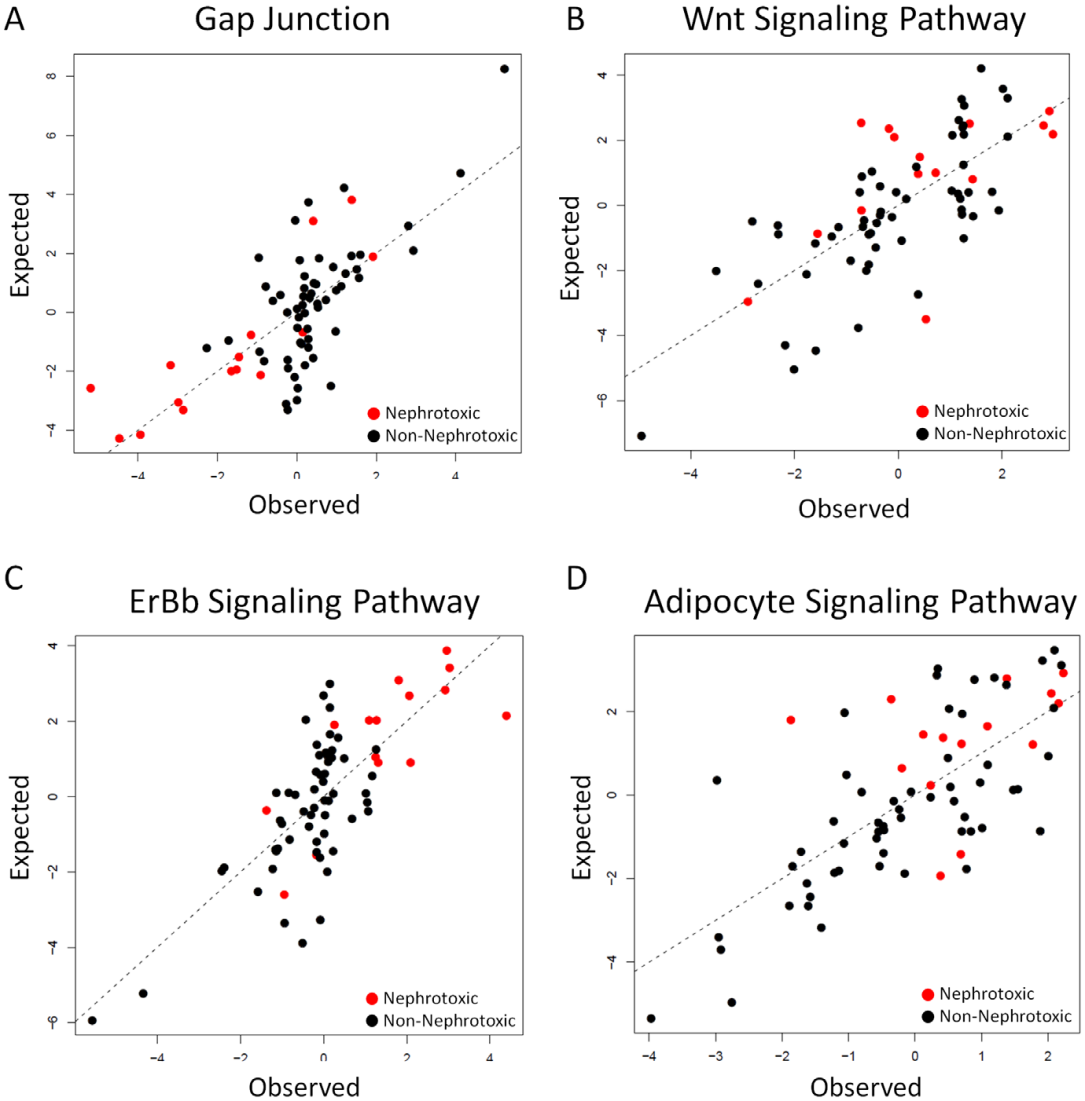
Example models linking PCFs with molecular pathway activity. The figure shows the relationships between the observed (x axis) and predicted (y axis) indices of pathway activity for a number of exemplar KEGG pathways. Nephrotoxic samples are represented by red dots whereas non-nephrotoxic samples are represented by black dots. *Gap Junction* and *ErbB Signaling Pathway* contain features belonging to ET-State indices, Geometrical descriptors and RDF descriptors. The 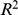 values are 0.55 and 0.57 respectively. Wnt Signaling Pathway and Adipocyte Signaling Pathway contain features belonging to GSFRAG, Information indices, Edge adjacency indices and 3D-MoRSE descriptors. The 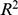 values are 0.52 and 0.51 respectively. Note that models containing a feature from E-State indices and RDF descriptors better separate nephrotoxic and non-nephrotoxic samples.

**Figure 6:**
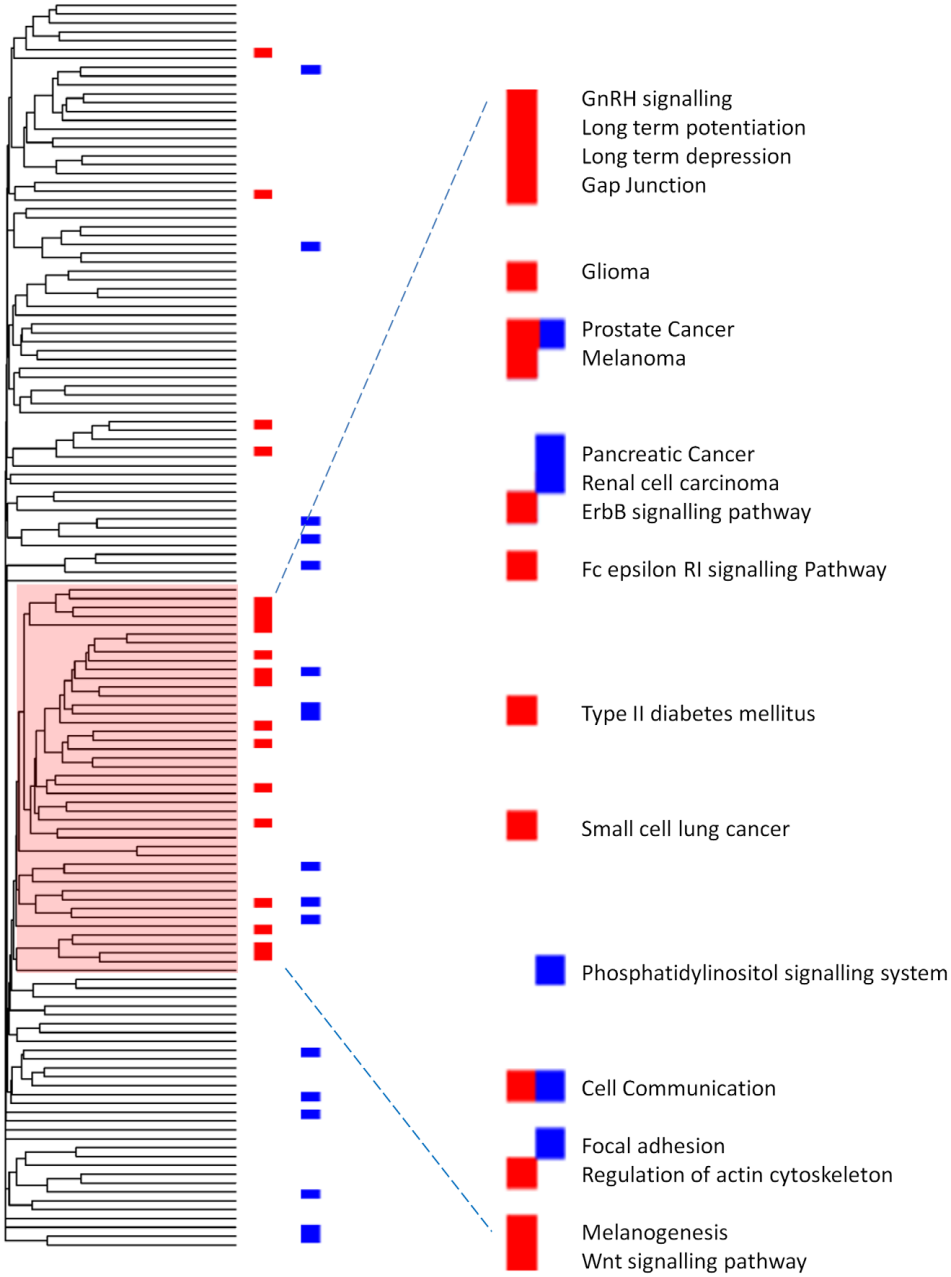
KEGG Pathway topology map. The Figure shows a dendrogram representing the degree of similarity between different KEGG pathways. Pathways marked in red are pathways that were found to be associated to chemical features (19), and pathways marked in blue have been found to be predictive of toxicity (21). Pathways whose activity is predicted by PCFs group in a tight cluster. Note that the majority of toxicity annotated pathways cluster towards the lower half of the dendrogram, close to pathways linked to PCFs.

Regression analysis described in the previous paragraph identified 19 pathways whose activity could be predicted by a combination of chemical features (See Figure 5 for some examples). Because of the apparent similarity in the molecular functions represented in these pathways we reasoned that these may be closely connected within the KEGG pathway map. In order to test this hypothesis we represented the relationship between individual pathways (defined by their degree of overlap) using hierarchical clustering. In this analysis KEGG pathways which share a larger number of components are represented in close proximity in the dendrogram. The visual inspection of the dendrogram confirmed that pathways, whose overall activity can be predicted by combinations of chemical features, were grouped in a compact cluster within the KEGG map (Figure 6). This cluster defines a KEGG super-pathway that represents a number of signalling networks directly connected to effectors functions of direct relevance with tissue morphogenesis such as Actin remodelling and *Cell communication*. Interestingly, *Cell communication* which we already mentioned to be associated to both PCFs and toxicity represents multiple signalling and effectors components of the cell to cell communication machinery. These include tight junction, gap junctions, adherence junctions, desmosomes and extracellular matrix components. The cluster of interconnected KEGG pathways defined by the association with chemical physical features therefore represents a network of signalling components which directly interact with a toxicity associated core of cell communication components.

### PCFs correlated to molecular pathway activity are best predictors of chemical induced toxicity

**Figure 7:**
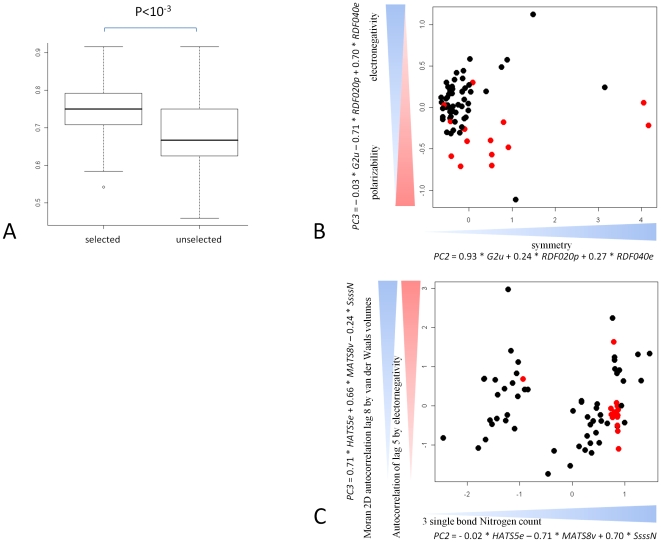
PCFs linked to molecular response are better predictors of toxicity. Panel A shows the comparison between the classification accuracy of models predictive of toxicity and developed selecting from PCFs which are predictive of molecular response and those developed using uncorrelated PCFs. Note that PCFs linked to molecular response have a higher predictivity (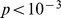). Panels B and C show the PCA representation of the samples using the 3 most represented features in the model populations. The information for the best separation in both instances is present in PC2 and PC3. The equations for panel B show that a high increase in symmetry and high polarizability and low electronegativity is predictive of toxicity. In the case of the unselected features panel C toxic chemicals do cluster together but are specific to containing a nitrogen with a triple single bond and a low autocorrelation.

The functional link between pathways associated to PCFs and toxicity may imply that the selected PCFs may be themselves predictive of renal tubular degeneration. In order to test this hypothesis we developed multivariate statistical models predictive of toxicity selecting from PCFs associated to molecular pathway activity. We then compared these with models developed from PCFs which where uncorrelated to the pathway associated PCFs subsets. Figure 7A shows that features predictive of molecular response are more predictive of toxicity outcome (average accuracy of 76% versus 68%, p-value 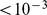). In order to identify the most representative PCFs subset, we developed representative models based on the three features which were most frequently represented in the model populations. Consistent with the previous result, the model built using PCFs associated to molecular response has higher sensitivity and specificity (sensitivity 0.781, specificity 0.871) than the one build with uncorrelated PCFs. This is reflected by a clearer sample separation in the PCA plot (Figure 7B). Features represented in the most predictive model combine two RDF descriptors and a WHIM descriptor whereas the unselected features model contains a GSFRAG, a GETAWAY descriptor and a 2D-autocorrelation descriptor. The model based on PCFs predictive of transcriptional response shows that toxic chemicals are characterized by high polarisability (RDF020p), low electronegativity (RDF040e) and low symmetry (G2u). Although the difference in accuracy (8%) is not particularly high, the results confirm that PCFs chosen by our method have a significantly higher ability to discern toxic from non-toxic chemicals.

## Discussion

**Figure 8:**
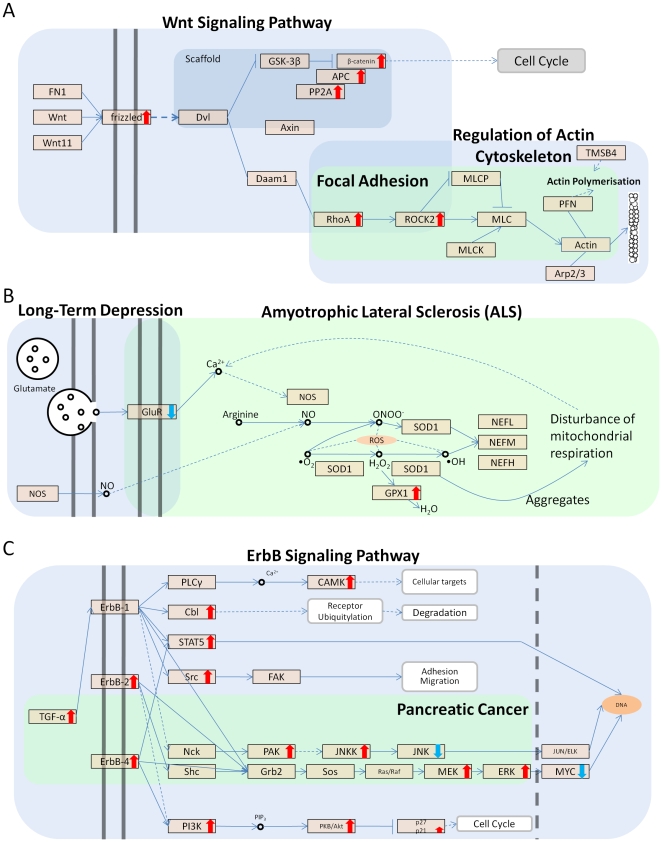
Association between PCFs and toxicity associated pathways. The figure represents the detailed relationship between pathways associated to chemical hits and pathways associated to toxicity. Pathways with membrane component were mostly associated to chemical hits whereas pathways with downstream signalling components were mostly associated to toxicity. This figure represents three possible links between pathways associated to chemical hits (*Wnt Signaling Pathway*, *Long-Term Depression* and *ErbB Signaling Pathway*) and toxicity (*Focal Adhesion*, *ALS* and *Pancreatic Cancer*) through shared genes between the pathways. Although each link presents a mechanism of action these were only implied by the pathway associated to toxicity. Genes found to be up or down regulated have been marked with a red or a blue arrow respectively.

The most important finding of our study is the demonstration that specific combinations of chemical descriptors can be predictive of the transcriptional activity of pathways, always using the second PC, representing the molecular state of a target organ after chemical exposure. These pathways (i.e. *ErbB Signalling*, *Wnt signalling*, *Long-Term Depression*, *Long-Term Potentiation* and several cancer pathways) mainly represent signalling pathways which in our model define the main domain of interaction between chemicals and cellular molecular response (Figure 8). We have shown that toxicity pathways with relevance to renal tubular degeneration are closely associated to this domain in the context of a KEGG pathway map. We explored close pathways by integrating the networks and establish whether, beyond the topological proximity, we could also identify a functional relationship between them. In this context we devised three interconnected pathways that could mechanistically explain the observed connection between chemical features, pathway activity and toxicity outcome. Figure 8A shows how a possible interaction between the *Wnt signalling pathway*, *Regulation of Actin Cytoskeleton* (linked to PCFs) and *Focal Adhesion* (predictive of toxicity outcome) could lead to a perturbation of actin cytoskeleton polymerization. More specifically, Wnt/Fz signalling activates the small GTPase Rho to control cell migration during tissue remodelling and development. This activation requires Dvl-Rho complex formation which is assisted by Daam1. From this it is clear that the integration of these topologically linked pathways represent a true series of biochemical events linking the binding of the Wnt ligand, through activation of Daam1 to the actin polymerization machinery. A plausible disturbance of mitochondrial respiration and energy balance by means of reactive oxygen species (ROS) generation is shown in Figure 8B. Lastly growth factor mediated modulation of the cell cycle, adhesion and cell migration through TGF-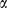 is shown in Figure 8C. This pathway module results from the integration of the *ErbB signalling pathway* (linked to PCFs) and *Pancreatic Cancer* (predictive of toxicity outcome). In this case the pathway linked to toxicity is a sub-network of the *ErbB signalling pathway* which represents the specific effects on tissue remodelling via regulation of cell growth, apoptosis and differentiation. The common feature among these hypothetical mechanisms is the association between chemical features and membrane associated cellular signalling and the large overlap between this and effectors pathways. Genes within each pathway are co-ordinately regulated across exposures suggesting that what we are modelling is not the effect of a small subset of highly regulated genes. Moreover, by mapping the direction of change between toxic and non-toxic chemicals on the KEGG pathway maps we observe that chemical exposure is associated to a coordinate overexpression of genes in signalling and effector genes (Figures S3, S4, S5, S6, S7, S8, S9, S10, S11, S12, S13, S14, S15, S16). It is therefore not unreasonable to hypothesize that the diverse spectrum of toxic chemicals used in this study may act via a general mechanism involving interaction with cellular membranes. This hypothesis is also consistent with the finding that polarisability is a key feature of the toxic chemicals studied (Figure 5 and 7). The interaction between chemicals and cellular membranes may perturb receptor signalling inducing changes in the expression of genes encoding for signalling components and ultimately creating an unbalance in the expression of effectors pathways involved in tissue dynamics and homeostasis. The regression models we built showed that, in many cases, there is a continuum of effects influencing the molecular state of a target pathway and that, in specific pathways, (i.e. *Gap junction* and *ErbB signalling pathways*) toxicity is observed either above or below a given threshold of pathway activity (Figure 5). This is showing that only chemicals that can substantially perturb key signalling pathways are able to induce stress responses such as disturbance of inter-cellular communication and mitochondrial disturbances that are frequently associated with subsequent cellular toxicity [18], [19]. It is possible that the proposed mechanism may be a general unifying mode of toxicity probably secondary to a range of initial specific mechanisms and that may act in parallel to the interaction with specific molecular targets. In this context, it is known that multiple and target-specific mechanisms of action of xenobiotics are responsible for drug induced nephropathy. For example, the targets of the initial insult may be at the level of altered blood flow, glomerular injury, direct proximal tubule damage or other tubule or papillary targets [20]. Furthermore nephropathy might be a direct action of the agent on nephrons or an indirect action such as via a reduction of prostaglandin production such as with salicylic acid, or via precipitation of liver-derived alpha-2-u-globulin as a result of chemical binding (e.g. d-limonene) [21]. Prominent as classes of nephrotoxic agents are halogenated hydrocarbons such as chloroform and bromobenzene and classes of therapeutic agents including nonsteroidal anti-inflammatory drugs, aminoglycosides and the anticancer agent cisplatin. These facts might suggest insurmountable difficulties in prediction of effects from structural characteristics because of a multiplicity of mechanisms. However, the focus of this paper is predominantly on agents that directly act on the tubular (principally proximal tubule) epithelial cells. Our study has shown that there are features of signalling disturbance that associate with both chemical structural parameters and also with additional molecular pathways that associate with toxicity. Integration of the datasets shows that it is possible to link structure to pathology via the two layers of analysis allowing a reconstruction of a series of pathways. The approach offers a new dimension to the existing strategies of databases that associate structure directly to known toxicity features through training (e.g. DEREK and TOPKAT [22] and the OECD Toolbox (www.oecd.org). The common signalling disturbance identified is thus hypothesised to lead to secondary effects linked to toxicity. It is the genome-wide surveillance strategy that has allowed the identification of the linkage which would not have been possible from more targeted analysis of individual mechanisms. Since the time point for the molecular changes observed is five days after exposure, it is also possible that the changes represent secondary intermediate modes of change rather than specific early mechanistic interactions. Interestingly, the modelled features associated with toxicity are not necessarily limited to nephrotoxicity. The biological implications of this work are further strengthened by the observation that chemical feature selection based on functional pathway activity leads to more predictive toxicity models (sensitivity 78.1%, specificity 87.1%). Therefore linking gene expression to chemical features identifies a sub-selection of features which are more linked to toxicity. We therefore propose that by integrating gene expression profiles with chemical feature information it may be possible to isolate a sub-group of features that are highly important in characterizing specific phenotypic effects allowing for a much better characterization of yet untested chemicals. The development of these methodologies is particularly important as large datasets representing a broader spectrum of chemicals are expected to become available. An excellent example of these publicly available datasets is the ToxCast™ [23] program which is currently running at the U. S. Environmental Protection Agency [24]. Several potential improvements may be necessary to make the approach fully generalizable. For example, the computation of pathway indices we have implemented is based on the use of PCs ensuring that a large percentage of variance (80% in this case) is retained. Although this is likely to work for most of the datasets, it is possible that PCA, which is based on a linear combination of variables, may not be able to capture more complex relationships with PCFs. Therefore it may be useful to consider other methods such as independent component analysis or a non-linear version of PCA. This issue is particularly important considering that in complex exposure experiments the component of variation associated to the interesting biological effect may be associated to non-specific effects of toxicity. It is therefore important that the procedure used for the construction of pathway indices has the potential to decompose these effects. However, even at the present stage of development, the broad application of the analysis strategy we have pioneered will improve our ability to identify mechanistic markers of toxicity and will help to better understanding the relationship between drug PCFs and cellular physiology.

## Methods

### The Dataset

The expression profiling dataset used in this analysis was originally developed by Iconix Biosciences [13]. It is at present the largest microarray-based analysis of chemical induced transcriptional response on a mammalian system being available in a public domain. In this study, rat kidneys have been profiled five days after exposure in a 28-day repeat-dose study in male Sprague-Dawley rats. The study involved 88 chemicals, 22 of which are known to induce renal tubular degeneration at the concentrations used in this study. Details of the experimental protocol are available in the original publication. Here we report a summary for clarity of the manuscript. Rats were treated daily and sacrificed on days 5 (n = 5 rats) and 28 (n = 10 rats) for kidney histopathology evaluation. Gene expression profiles were obtained on day 5 from 3 randomly chosen rats per treatment group, before the expected appearance of the lesions. Doses were chosen so as to not cause histological or clinical evidence of renal tubular degeneration after 5 days of dosing, but to cause late-onset histological evidence of tubular degeneration as expected from the literature. The negative class of this dataset was defined based on literature knowledge of treatment effects in humans and rodents. This class included 49 non-nephrotoxic compound treatments that were administered daily for 5 or 7 days (n = 3 rats). The dose was an empirically determined maximum tolerated dose in order to ensure sufficient exposure, but not to cause overt clinical toxicity. This was defined as the dose that causes approximately a 50% decrease in body weight gain relative to controls during the course of the 5-day range finding study, and without severe clinical signs of toxicity.

### Summarizing the transcriptional state of kidney using indices of pathway transcriptional activity

In order to reduce the complexity of linking chemical descriptors to the kidney transcriptional state we have computed indices of overall pathway transcriptional activity [25], [26]. These indices were computed by mapping the 7478 genes represented in the pre-processed dataset [13] onto KEGG pathways. In choosing the number of PCs we have used the simple criteria of selecting subsequent components to explain at least 80% of the variance. In this dataset this lead to the selection of the first two components. Using this criteria we summarized the activity of pathways including more than 5 genes (148) by computing the first two principal components (PCs) which were always able to summarize up to 89% of the variance. The advantage of using PCs is that the inter-gene correlation structure is automatically incorporated into the process of dimension reduction, so this information is not lost. Computation of the PCs has been performed using the principal component function prcomp within the software programming environment R [27].

### Comparing indices of pathway activity in response to chemical exposure

In this analysis we have compared indices of pathway activity between treated samples and matched controls (Figure 1, Step 2) and between toxic and non-toxic chemicals (Figure 2, Step 2). In both cases significantly differentially modulated pathways have been identified by a combination of dimension reduction via Principal Coordinates with Hotelling's multivariate extension of the t-test. Versions of this approach were independently developed by Kong et al. [28] and Song et al. [29], and made available in the R Bioconductor package pcot2 [29]. As mentioned in the previous paragraph, one of the advantages of using PCs is the fact that the inter-gene correlation structure is incorporated into the process of dimension reduction. The Hotelling's 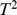 procedure, applied to both pathway components allows this correlation to be included in the test statistic for each pathway modules [30]. In the first case the output of the 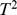 Hotelling's test has been used as an index of drug effectiveness to perturb the homeostatic state (Figure 1, Step 2). Indices have then been used as inputs in a hierarchical clustering procedure to compare drugs perturbation profiles. In order to identify which PC most contributes to the separation a univariate t-test has been applied to the first and second PC separately and the resulting dendrograms compared (Figure 3). In the second case the 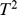 Hotelling's test has been used to identify pathways that are differentially modulated between toxic and non-toxic chemicals (Figure 2, Step 2). The p-values obtained from this test were corrected for multiple testing using the Benjamini and Hochberg method [31]. Pathways with an FDR
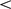
1% were considered differentially active between the two experimental groups.

### Deriving chemical physical features (PCFs)

PCFs were computed using the Web-based toolset e-dragon [32]. E-dragon computes 2352 chemical descriptors by integrating several publicly available methodologies. Only features that could be computed for all chemicals in the dataset were used leading to a total of 1515 chemical physical descriptors (Dataset S1).

### Linking chemical features to pathway activity components

In order to link chemical descriptors to a given pathway component we used a regression model based on the combination of three chemical descriptors, including interaction components (Equation 1). More precisely, we define:
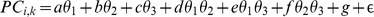
(1)Where 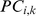 is the principal component i of pathway k. 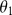, 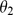 and 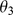 are three different given chemical descriptors, a, b, c, d, e, f, g are model parameters and 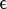 is the noise model component. In order to select an optimal subset of chemical descriptors we have used a genetic algorithm (GA) based methodology as implemented in the R package GALGO [33]. We used this random search procedure to find an optimal sub-set of variables to maximize the model 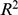 value. In this application, data where split in training (2/3 of the samples) and test (1/3 of the samples) sets. The training set was used as an input to the GA procedure to search for predictive models. The fitness function was implemented as a linear model denoted in (1). The fitness value for model selection was set to 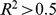. To estimate the 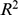 accurately a 5-fold cross validation procedure was used. The GA procedure was then allowed to run for 1000 simulations. Pathways for which we could identify predictive models were considered for further analysis. This resulted in the identification of 19 pathways linked to PCFs (Table 2, Table S3 for further details). Figure 5 shows examples of models found by the GA in which the predicted values using an optimized model are plotted against the observed PC values for a given pathway.

**Table 2:** This table lists the 19 pathways whose activity could be predicted by combinations of PCFs.

KEGG ID	Pathway	R^2^ average	R^2^	Feature Type		
rno04810	Regulation of actin cytoskeleton	0.53	0.53	GETAWAY descriptors	GETAWAY descriptors	ET-state Indices
rno05222	Small cell lung cancer	0.52	0.57	RDF descriptors	GSFRAG Descriptor	GETAWAY descriptors
rno04310	Wnt signaling pathway	0.53	0.57	GSFRAG Descriptor	Information indices	GSFRAG Descriptor
rno04540	Gap junction	0.53	0.57	Geometrical descriptors	ET-state Indices	3D-MoRSE descriptors
rno04720	Long-term potentiation	0.53	0.57	GETAWAY descriptors	Information indices	GSFRAG Descriptor
rno04730	Long-term depression	0.53	0.54	RDF descriptors	ET-state Indices	Geometrical descriptors
rno04912	GnRH signaling pathway	0.52	0.55	GETAWAY descriptors	WHIM descriptors	WHIM descriptors
rno04916	Melanogenesis	0.52	0.53	3D-MoRSE descriptors	Geometrical descriptors	ET-state Indices
rno00860	Porphyrin and chlorophyll metabolism	0.52	0.53	ET-state Indices	GSFRAG Descriptor	WHIM descriptors
rno00010	Glycolysis/Gluconeogenesis	0.52	0.53	GSFRAG Descriptor	GSFRAG Descriptor	WHIM descriptors
rno01430	Cell Communication	0.52	0.57	Geometrical descriptors	ET-state Indices	GETAWAY descriptors
rno04920	Adipocytokine signaling pathway	0.51	0.53	GSFRAG Descriptor	GSFRAG Descriptor	2D autocorrelations
rno04012	ErbB signaling pathway	0.53	0.57	RDF descriptors	ET-state Indices	Geometrical descriptors
rno05215	Prostate cancer	0.52	0.53	RDF descriptors	GSFRAG Descriptor	GETAWAY descriptors
rno04664	Fc epsilon RI signaling pathway	0.53	0.55	3D-MoRSE descriptors	GSFRAG Descriptor	2D autocorrelations
rno05214	Glioma	0.52	0.55	3D-MoRSE descriptors	Molecular properties	Topological charge indices
rno05218	Melanoma	0.51	0.59	ET-state Indices	3D-MoRSE descriptors	Geometrical descriptors
rno04930	Type II diabetes mellitus	0.53	0.57	WHIM descriptors	GSFRAG Descriptor	GSFRAG Descriptor
rno00480	Glutathione metabolism	0.51	0.54	Edge adjacency indices	Information indices	3D-MoRSE descriptors

For each pathway the average R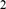 value given the model population, the model with the highest R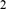 value and the descriptor group features responsible for the correlation are shown. A detailed table with the features that have been selected for these models can be found in Table S3.

### Creating and visualizing a KEGG pathway map

In order to visually represent the relationship between the different KEGG pathways we computed a pathway similarity matrix based on the Jaccards Index of overlap. This is defined as the ratio between the numbers of genes shared by any two pathways (intersection) divided by the number of unique genes in the two combined pathways (union). The resulting matrix was used as an input to a hierarchical clustering procedure (average linkage). The effectiveness of the clustering procedure in representing the information described by the similarity matrix has been verified using the cophenetic function correlation fit to the input overlap matrix (r = 0.9).

### Predicting renal tubular degeneration from chemical descriptors

Different subsets of chemical features were used to develop multivariate predictors of chemical toxicity using a classis QSAR methodology. The first subset (92 features) was defined including descriptors represented in the models predictive of pathway activity whereas the second subset included all variables not selected in the predictive models and that were uncorrelated, an absolute pearson coefficient of less than 0.5, to PCFs from the first group (210 features). Models were developed using a maximal likelihood discriminant function coupled to a genetic algorithm for variable selection using default settings [33]. A forward selection approach was used to identify the single smallest model, with the least number of descriptors and with the highest classification accuracy [33]. Classification accuracy was estimated using a k-folds cross-validation procedure. Interpretation of the models has been performed with the help of PCA.

## Supporting Information

Figure S1 PC1 and PC2 relationship to toxicity. A) PCA scatterplot of the chemical space using all genes clustered into KEGG Pathways. Chemicals marked black or red are nephrotoxic and non-nephrotoxic respectively. B) Boxplot showing the separation on the second component between nephrotoxic and non-nephrotoxic chemicals. A t-test between the two sets has a p-value <0.001. C) Dose separation on the PCA plot. Low-dose chemicals are marked in red, and high-dose chemicals in black. We observe a diagonal relationship between PC1 and PC2 separating the dose. More specifically, as shown in (a) the toxic chemicals separate on the 2nd PC. This implies that part of the non-toxic dose component is summarized in PC1.
(0.38 MB TIF)

Figure S2 Distribution of interaction components. The figure shows the distribution of interaction components for the 19 pathways found to be associated to chemical features. It can be seen that most of the interaction components add more than 50% towards the resulting model.
(0.03 MB TIF)

Figure S3 Heatmap of the genes belonging to Amyotrophic lateral sclerosis (ALS).
(2.19 MB TIF)

Figure S4 PC2 loadings of the genes belonging to the Amyotrophic lateral sclerosis (ALS).
(0.01 MB TIF)

Figure S5 Heatmap of the genes belonging to regulation of actin cytoskeleton.
(3.06 MB TIF)

Figure S6 PC2 loadings of the genes belonging to regulation of actin cytoskeleton.
(0.02 MB TIF)

Figure S7 Heatmap of the genes belonging to ErbB signaling pathway.
(3.51 MB TIF)

Figure S8 PC2 loadings of the genes belonging to ErbB signaling pathway.
(0.01 MB TIF)

Figure S9 Heatmap of the genes belonging to focal adhesion.
(4.23 MB TIF)

Figure S10 PC2 loadings of the genes belonging to focal adhesion.
(0.02 MB TIF)

Figure S11 Heatmap of the genes belonging to long-term depression.
(2.38 MB TIF)

Figure S12 PC2 loadings of the genes belonging to long-term depression.
(0.01 MB TIF)

Figure S13 Heatmap of the genes belonging to pancreatic cancer.
(3.77 MB TIF)

Figure S14 PC2 loadings of the genes belonging to pancreatic cancer.
(0.01 MB TIF)

Figure S15 Heatmap of the genes belonging to Wnt signaling pathway.
(3.63 MB TIF)

Figure S16 PC2 loadings of the genes belonging to Wnt signaling pathway.
(0.02 MB TIF)

Table S1 Representation of KEGG pathways. This table shows the KEGG pathways represented in this dataset and their relative sizes.
(0.05 MB XLS)

Table S2 KEGG pathway occurency table. This table shows the percentage of KEGG pathways found in each high level pathway category in the KEGG Pathway Database.
(0.02 MB XLS)

Table S3 Detailed model description. Here, we show a detailed description of a model for each pathway with the highest R^2^ value.
(0.04 MB XLS)

Dataset S1 Supplementary dataset.
(2.01 MB CSV)
