# Supplementary figures and images for: Mapping Drug Physico-Chemical Features to Pathway Activity Reveals Molecular Networks Linked to Toxicity Outcome
Source: PLoS One. 2010 Aug 27;5(8):e12385. doi: 10.1371/journal.pone.0012385 (PMC2929951; doi:10.1371/journal.pone.0012385)

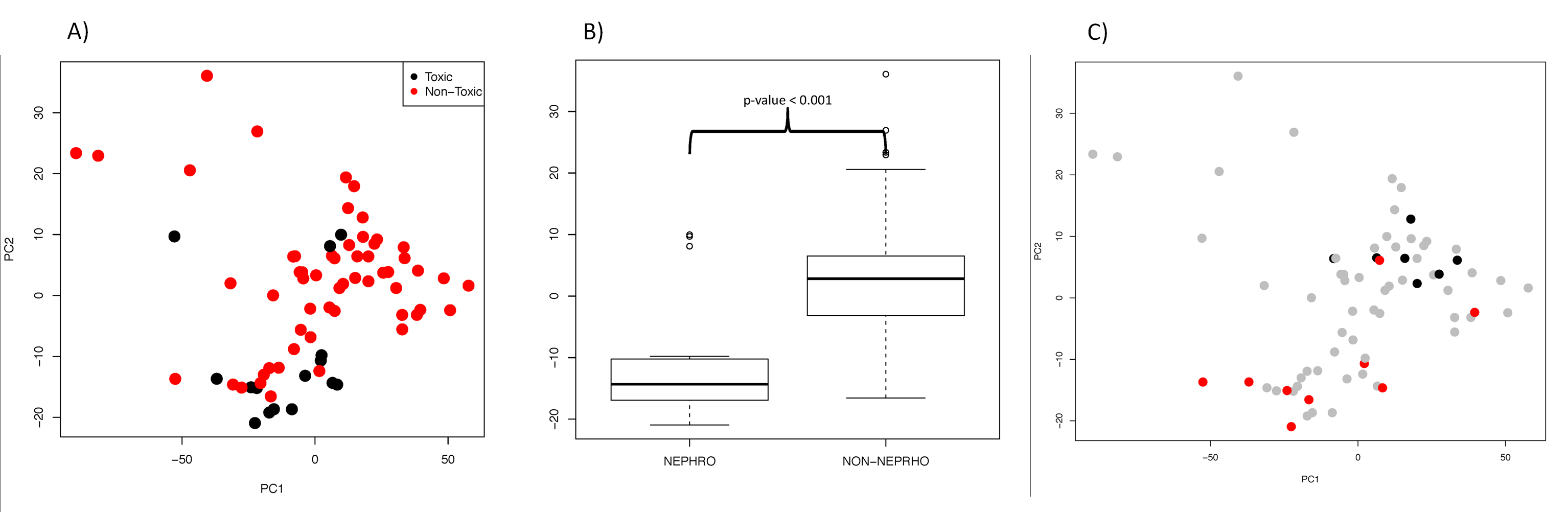

Supplement: Figure S1 — PC1 and PC2 relationship to toxicity. A) PCA scatterplot of the chemical space using all genes clustered into KEGG Pathways. Chemicals marked black or red are nephrotoxic and non-nephrotoxic respectively. B) Boxplot showing the separation on the second component between nephrotoxic and non-nephrotoxic chemicals. A t-test between the two sets has a p-value <0.001. C) Dose separation on the PCA plot. Low-dose chemicals are marked in red, and high-dose chemicals in black. We observe a diagonal relationship between PC1 and PC2 separating the dose. More specifically, as shown in (a) the toxic chemicals separate on the 2nd PC. This implies that part of the non-toxic dose component is summarized in PC1. (0.38 MB TIF) [file pone.0012385.s001.tif]

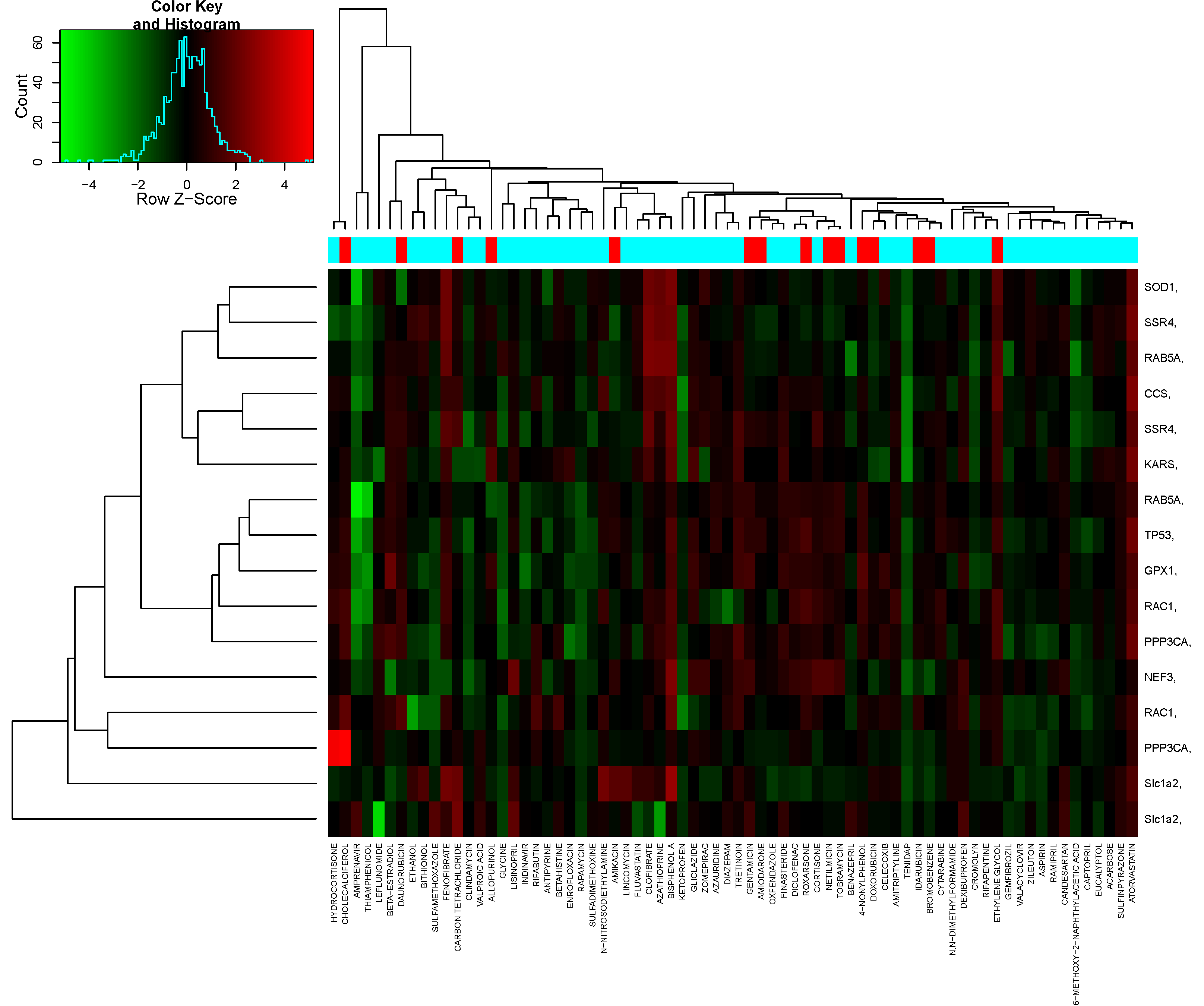

Supplement: Figure S3 — Heatmap of the genes belonging to Amyotrophic lateral sclerosis (ALS). (2.19 MB TIF) [file pone.0012385.s003.tif]

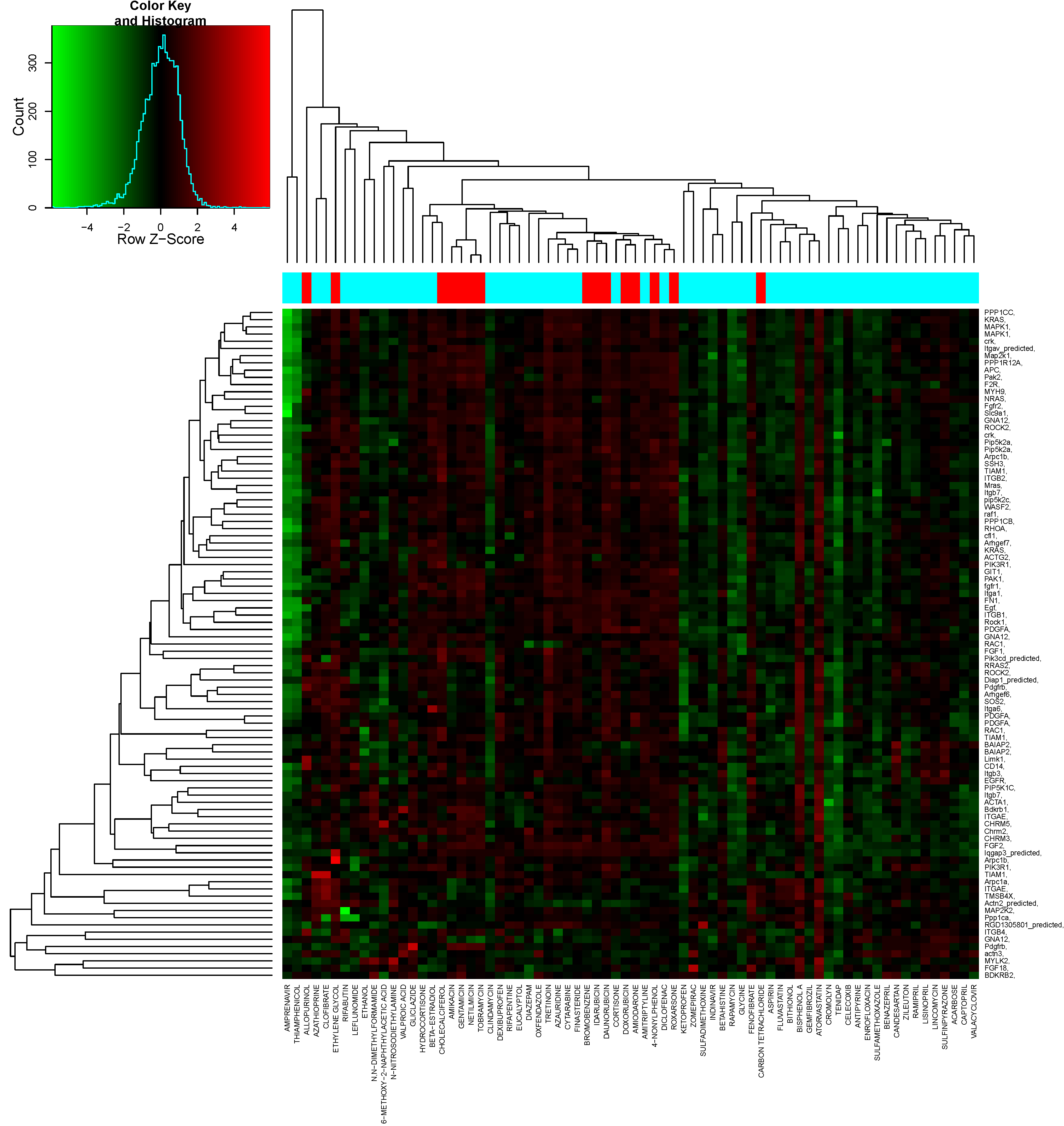

Supplement: Figure S5 — Heatmap of the genes belonging to regulation of actin cytoskeleton. (3.06 MB TIF) [file pone.0012385.s005.tif]

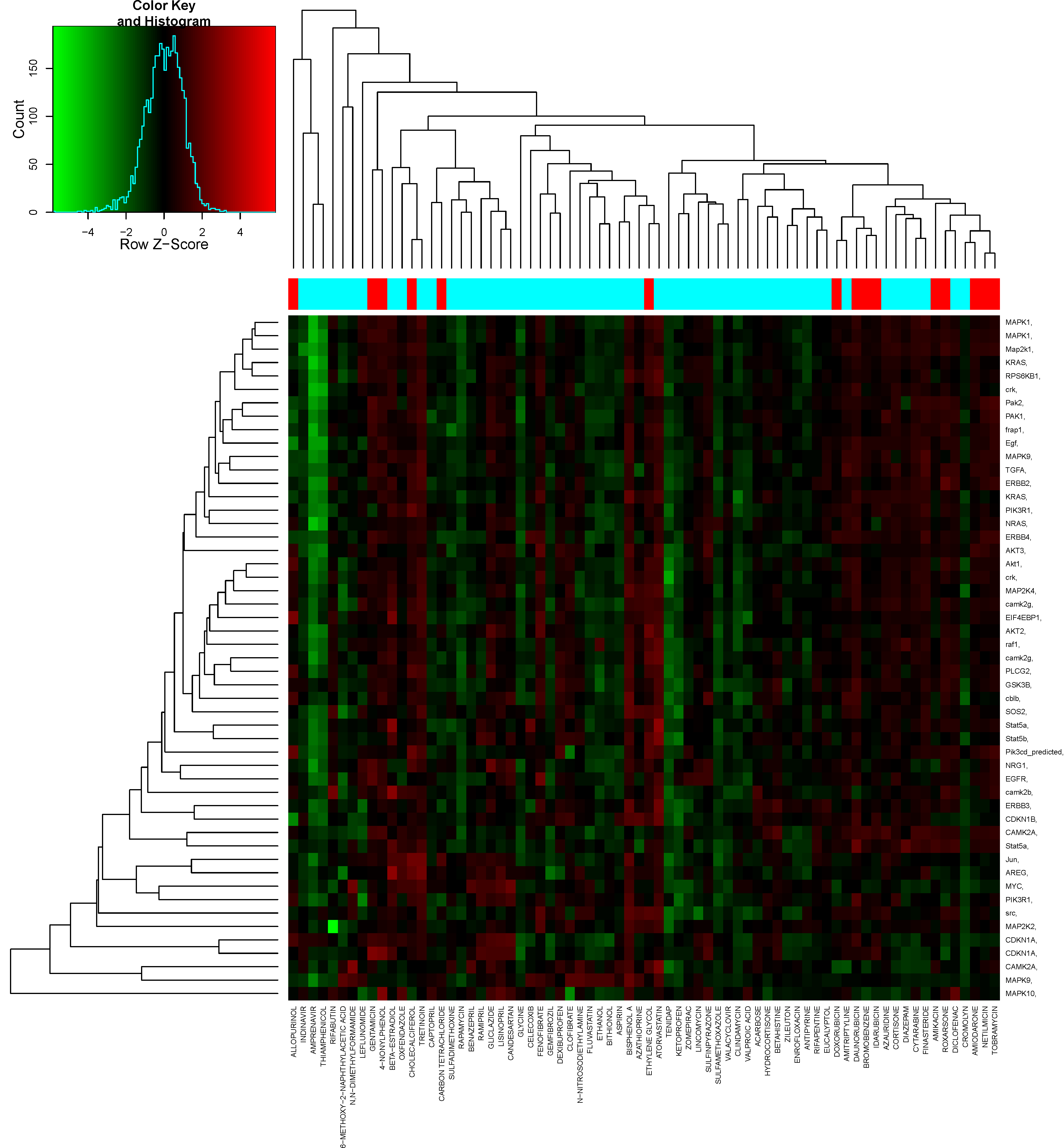

Supplement: Figure S7 — Heatmap of the genes belonging to ErbB signaling pathway. (3.51 MB TIF) [file pone.0012385.s007.tif]

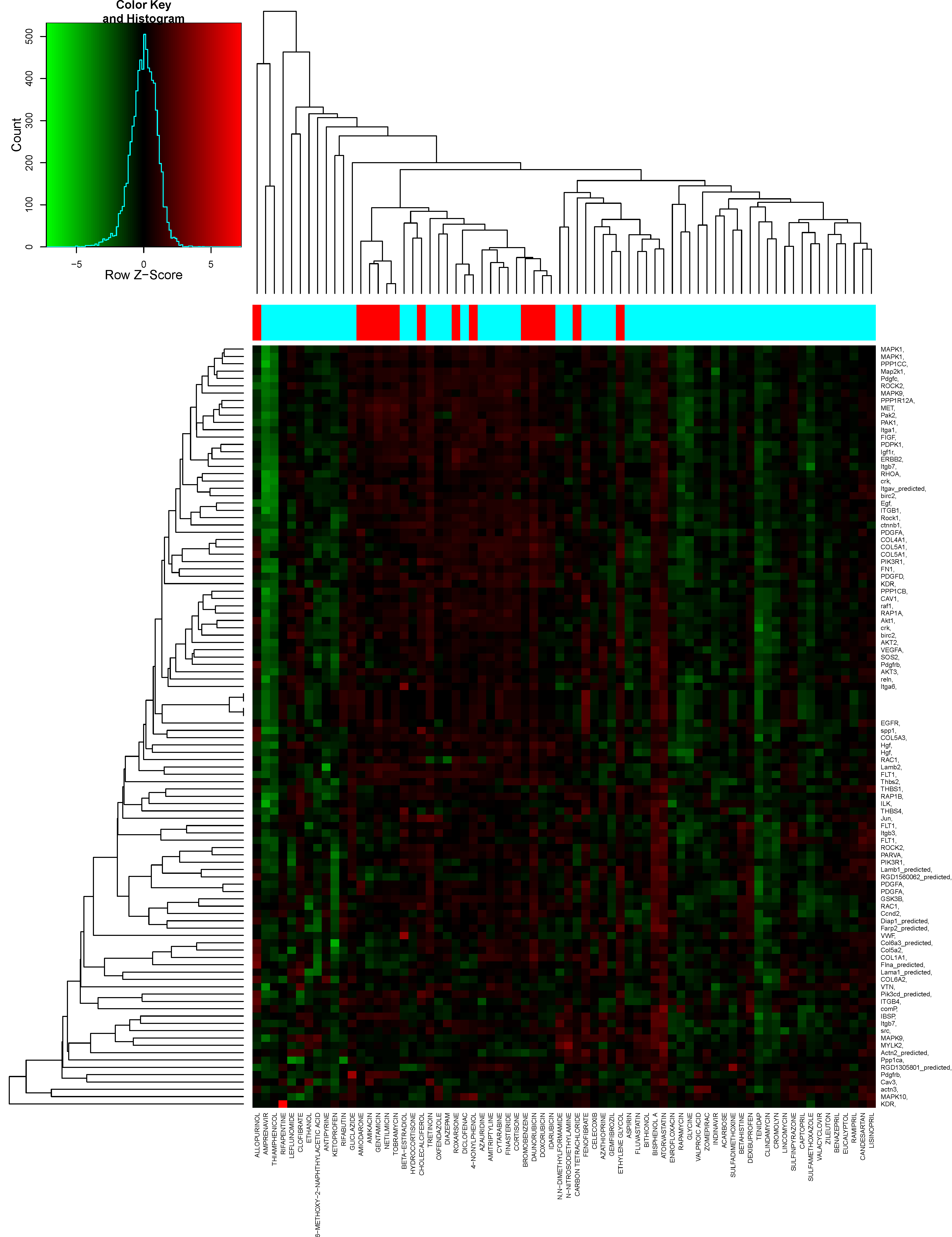

Supplement: Figure S9 — Heatmap of the genes belonging to focal adhesion. (4.23 MB TIF) [file pone.0012385.s009.tif]

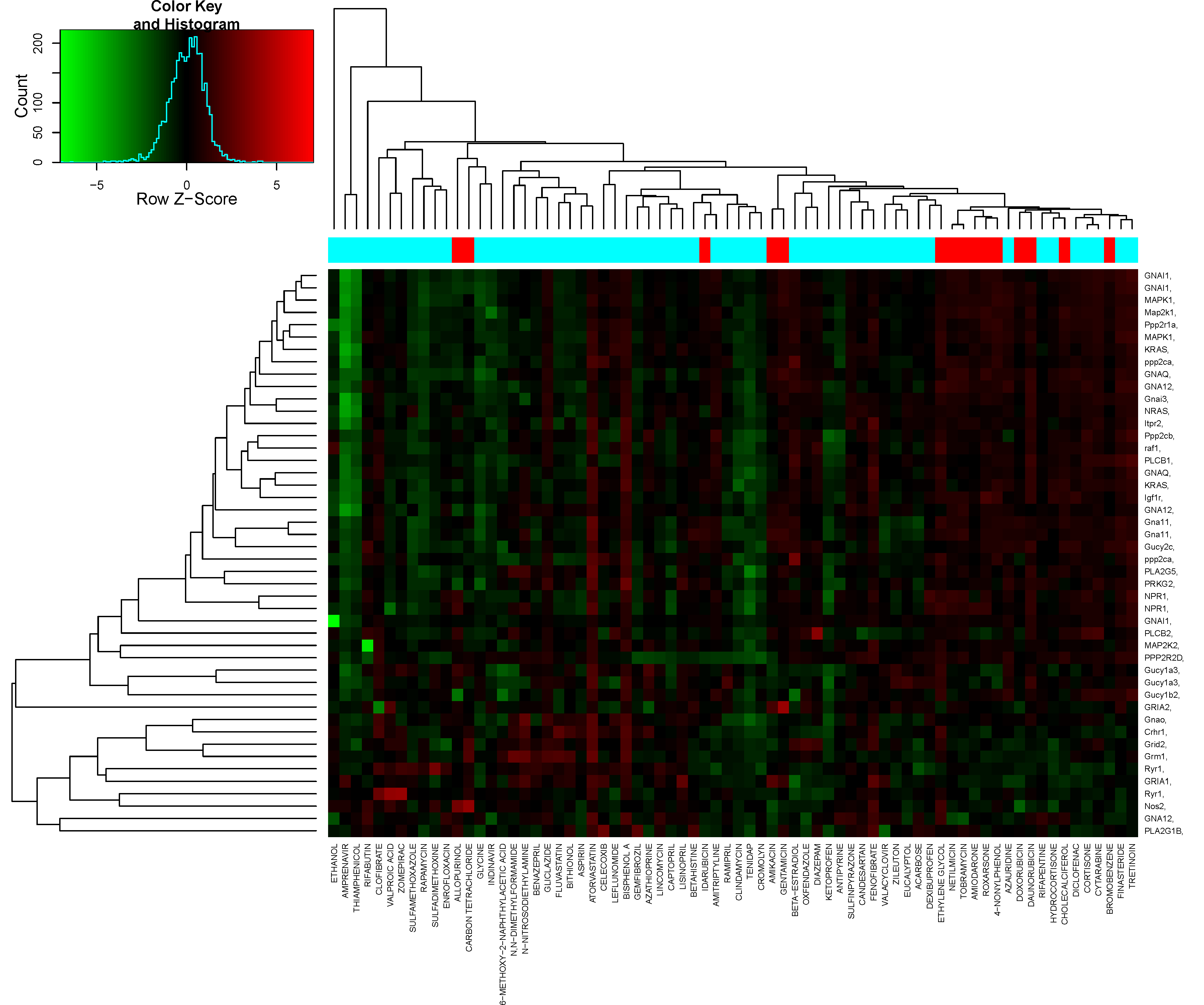

Supplement: Figure S11 — Heatmap of the genes belonging to long-term depression. (2.38 MB TIF) [file pone.0012385.s011.tif]

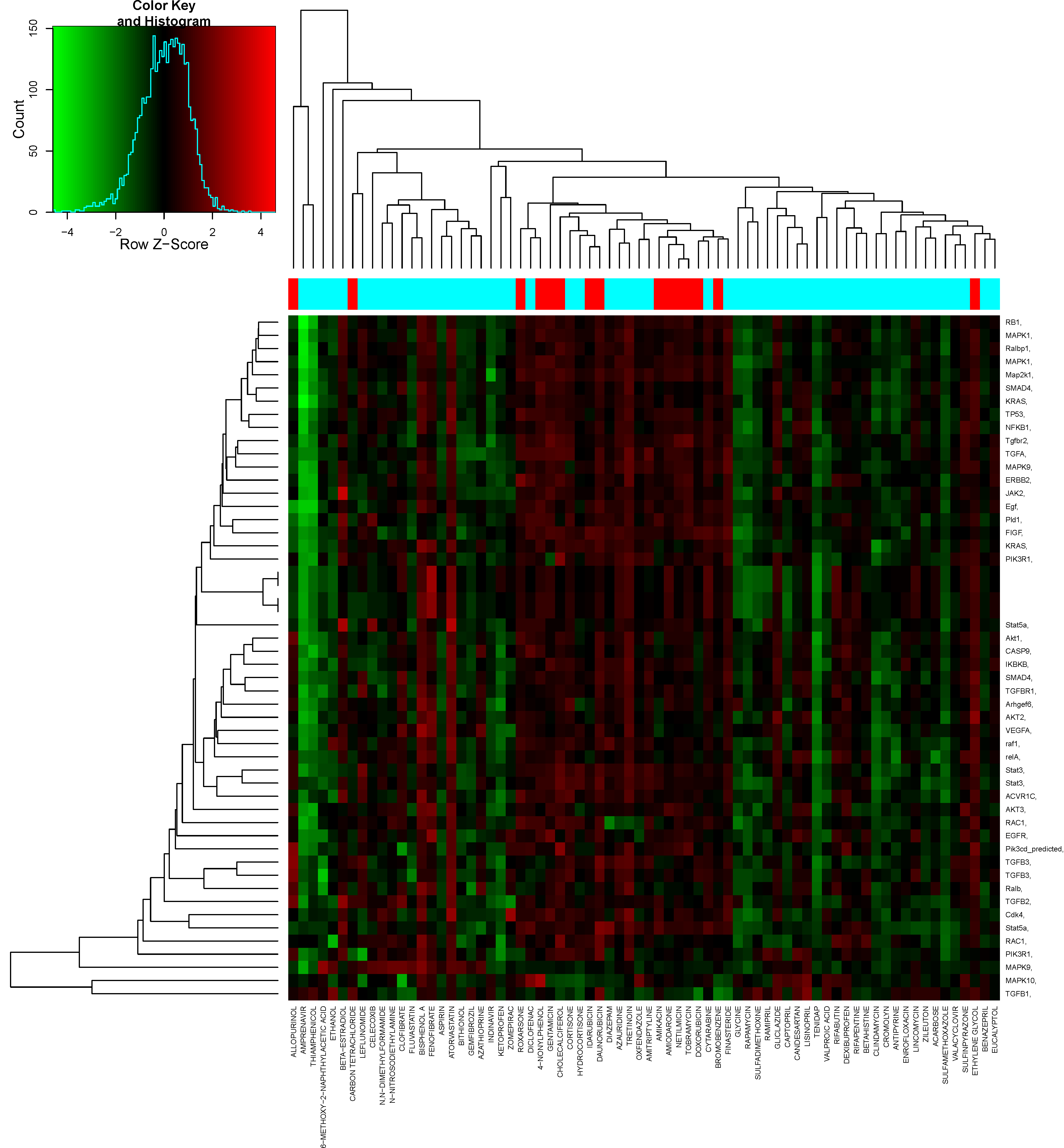

Supplement: Figure S13 — Heatmap of the genes belonging to pancreatic cancer. (3.77 MB TIF) [file pone.0012385.s013.tif]

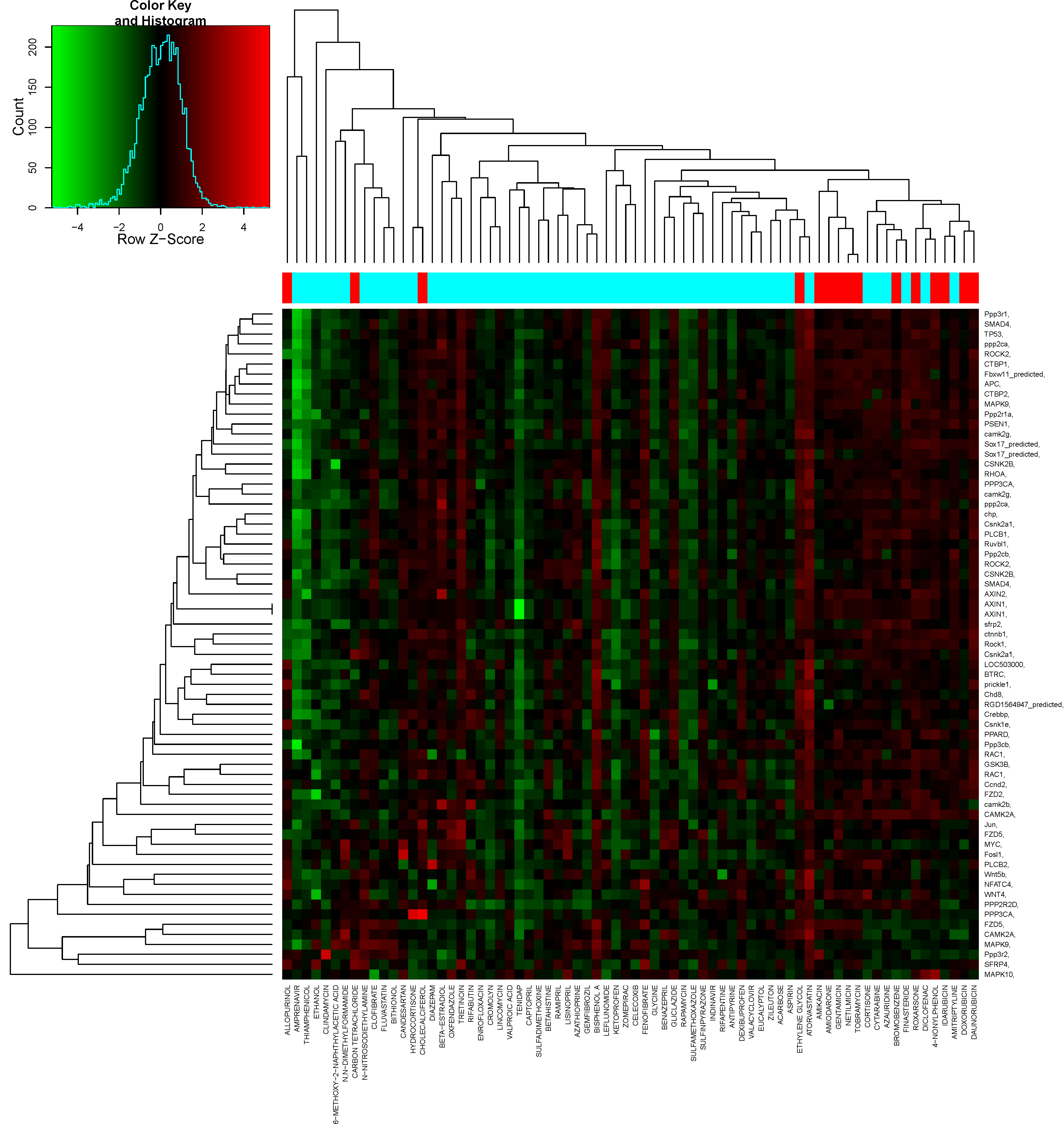

Supplement: Figure S15 — Heatmap of the genes belonging to Wnt signaling pathway. (3.63 MB TIF) [file pone.0012385.s015.tif]
